# Undercover lung damage in pediatrics - a hot spot in morbidity caused by collagenoses

**DOI:** 10.3389/fimmu.2024.1394690

**Published:** 2024-06-27

**Authors:** Ancuta Lupu, Maria Oana Sasaran, Elena Jechel, Alice Azoicai, Monica Mihaela Alexoae, Iuliana Magdalena Starcea, Adriana Mocanu, Alin Horatiu Nedelcu, Anton Knieling, Delia Lidia Salaru, Stefan Lucian Burlea, Vasile Valeriu Lupu, Ileana Ioniuc

**Affiliations:** ^1^ Mother and Child Medicine Department, “Grigore T. Popa” University of Medicine and Pharmacy, Iasi, Romania; ^2^ Faculty of Medicine, “George Emil Palade” University of Medicine, Pharmacy, Science and Technology, Targu Mures, Romania; ^3^ Faculty of Medicine, “Grigore T. Popa” University of Medicine and Pharmacy, Iasi, Romania; ^4^ Public Health and Management Department, “Grigore T. Popa” University of Medicine and Pharmacy, Iasi, Romania

**Keywords:** systemic lupus erythematosus, scleroderma, juvenile dermatomyositis, pulmonary damage, physiopathological mechanisms, overlap syndrome, child

## Abstract

Connective tissue represents the support matrix and the connection between tissues and organs. In its composition, collagen, the major structural protein, is the main component of the skin, bones, tendons and ligaments. Especially at the pediatric age, its damage in the context of pathologies such as systemic lupus erythematosus, scleroderma or dermatomyositis can have a significant negative impact on the development and optimal functioning of the body. The consequences can extend to various structures (e.g., joints, skin, eyes, lungs, heart, kidneys). Of these, we retain and reveal later in our manuscript, mainly the respiratory involvement. Manifested in various forms that can damage the chest wall, pleura, interstitium or vascularization, lung damage in pediatric systemic inflammatory diseases is underdeveloped in the literature compared to that described in adults. Under the threat of severe evolution, sometimes rapidly progressive and leading to death, it is necessary to increase the popularization of information aimed at physiopathological triggering and maintenance mechanisms, diagnostic means, and therapeutic directions among medical specialists. In addition, we emphasize the need for interdisciplinary collaboration, especially between pediatricians, rheumatologists, infectious disease specialists, pulmonologists, and immunologists. Through our narrative review we aimed to bring up to date, in a concise and easy to assimilate, general principles regarding the pulmonary impact of collagenoses using the most recent articles published in international libraries, duplicated by previous articles, of reference for the targeted pathologies.

## 1 Introduction

Connective tissue diseases (CTDs) that frequently affect pediatric patients are systemic lupus erythematosus (SLE), scleroderma (Sc) and dermatomyositis (DM). In more particular cases, we can find antiphospholipid antibody syndrome, Sjögren's syndrome or associations of pathologies (overlap syndrome). The difficulty of approaching the cases lies both in the variety of clinical manifestations of the underlying disease, as well as in the multiple systemic damages that can occur (e.g., cerebral, pulmonary, cardiac, renal) (1). Regarding pulmonary damage, the pathogenic mechanisms can be diverse starting from granulomatous reaction, interstitial inflammation, primary vasculitis and ending with disease mediated by immune complexes. The functional imbalance can be objectified equally in patients who do not present pulmonary imaging changes (2). Consequently, given the impact played by pulmonary pathology on the patient's quality of life, understanding the pathological mechanisms underlying them, early detection and countering/slowing down the decline represent the goals of modern, preventive medicine.

The current narrative review aims to outline the most current medical information regarding the pathophysiological cascade underlying lung degradation in CTD. In this sense, we will consider a concise presentation regarding the main respiratory damage that can occur in the evolution of the pediatric patient with collagenoses. Additionally, we will not omit from the discussion the current standards of their diagnosis and management. Finally, we will combine the results certified in the literature with those currently being researched, thus broadening the horizons of approaching pediatric children with collagenoses from the perspective of respiratory system disorders. The desired practical purpose is to obtain an increase in the quality of life by drawing attention to the chameleon-like ways of presentation, encouraging early detection and appropriate, individualized management. In order to achieve our objectives, we performed a screening of the most recent publications in the field of interest. We have added reference articles to these. The databases entered were PubMed, Google Scholar, Web of Science, Scopus and Embase. No linguistic criteria were imposed, in order to limit the risk of bias. The terms used for the search were "pulmonary disease", "collagenosis", "collagenoses", "systemic lupus erythematosus", "scleroderma", "dermatomyositis", "children" and various combinations thereof.

## 2 Current directions in knowledge

### 2.1 General considerations

CTDs are thus broadly represented by the triad SLE – Sc – DM. Although the characteristics at the pediatric age are slightly different from those of adults, their importance cannot be neglected from the perspective of the increased morbidity and mortality that they print.

SLE is a multisystemic autoimmune condition, with a pathophysiology based on genetic and epigenetic factors and a disabling evolution. The genetic involvement is demonstrated by recent studies regarding the increased susceptibility to develop the juvenile form of SLE (jSLE) among children with positive mothers for SLE or Sjögren's syndrome. Compared to the adult form, the juvenile onset is marked by a higher activity of the disease, important organic lesions, and significant therapeutic burden. The aggressiveness of the disease is inversely proportional to the age at diagnosis, under 5 years of age atypical manifestations of the disease can also be observed (e.g., lack of autoantibodies). Therefore, an overall mortality rate of about three times the usual rate is estimated (3–5). Due to these considerations, the medical world is focusing its efforts on the discovery of new biomarkers (blood/urine) useful in the diagnosis, monitoring of jSLE, and predicting the response to treatment (6, 7). It is also worth mentioning the existence of a neonatal form of SLE that occurs in the case of transplacental transfer of the maternal antigen. The clinical manifestations are violent, including skin, cardiac, hepatobiliary, and hematological damage and regressing (with the exception of cardiac symptoms) once the antibodies are cleared (8). Representing up to 15% of the total number of cases, jSLE notes an incidence that varies between 0.36–2.5/100,000 children, with a prevalence of 1.89–34.1/100,000 children. It is noted that most patients have an onset of jSLE in peri-puberty/adolescence. Regarding the racial distribution, jSLE appears to present more classic clinico-biological characteristics among African/Caribbean patients compared to Caucasian patients. On the other hand, the renal damage was more pronounced in the first category, reaching more frequent therapies with Cyclophosphamide and Rituximab. The gender distribution is in favor of girls, who are affected up to ten times more often than boys (3, 9). Organic damage is an invariable and apparently multifocal phenomenon in the evolution of SLE. Factors such as demographic characteristics, duration of the disease and age at onset, genetic predisposition, the presence of antiphospholipid antibodies or pharmacotherapy appear to play a role in its initiation and maintenance (10). Recent theories also place in the area of interest the impact played by sensitization due to infections (e.g., Covid 19, Epstein-Barr virus) or the imbalance of the microenvironment (e.g., dysbiosis, nutritional/immune deficiency, atopic terrain, hormonal or neuro-psychic disturbances) (11–14). The therapeutic protocol is largely based on the adaptation of the one used among adult patients (7). Thus, considering the stated conditions, it is seen necessary to undertake global efforts aimed at preventing and reducing the negative impact of jSLE on the quality of life.

Juvenile scleroderma (jSc) is considered the third most common rheumatic disease in childhood. Clinically, it gathers under its umbrella manifestations such as inflammation, vasculopathy and fibrosis. According to the degree of its damage, it can be localized (jLSc) or systemic (jSSc). Although jSc is primarily considered a localized disease, the results obtained by Zulian F. et al. contradict the paradigm. They expose the possibility of single or multi-organ damage within jSc, without increasing the risk of progression to jSSc. Consequently, a thorough and extensive evaluation of the patients is required. In turn, the systemic form is divided into diffuse cutaneous (diffuse and rapidly progressive thickening of the skin that associates early pulmonary, cardiac or renal damage), limited cutaneous (restricted and non-progressive thickening of the skin, limited to the distal extremities, which associates late pulmonary arterial hypertension or malabsorption) and overlap syndrome that may associate features of another connective tissue disease (e.g., dermatomyositis, SLE). Respiratory, the difference between the 3 forms is dictated by the more frequent cardiac damage in the skin-limited one and pulmonary in the others. Regardless of the form we are dealing with, it should be known that up to 1/3 of scleroderma cases begin in childhood. The average age of diagnosis is 6–8 years (jLSc), and respectively 8–11 years (jSSc). The incidence varies between forms from 0.34–2.7/100,000 children/year in the localized form, to 0.27/1,000,000 children/year in the systemic one. The gender ratio is against girls in both forms, while the racial predominance of jLSc is higher among Caucasians (15–19). The organic damage seems to be similar between children and adults. However, compared to the adult form, the prognosis in the medium and long term (5 to 15 years) is more favorable in the juvenile form. However, the data must be interpreted with caution due to the disproportionality of the compared cohorts. Martini G. et al. also mentions the reverse side of the coin, represented by the existence of a form with very rapid progression and early signs of internal organ involvement. There are also rare reports of apparently jLSc forms that have progressed to jSSc. The main factors influencing mortality are damage to vital organs such as the heart (pericarditis), kidneys (increase in creatinine) and lungs (pulmonary fibrosis) (17, 20–23).

Juvenile dermatomyositis (jDM) is the last collagenoses disease on which we focus our attention. The onset of vasculopathy is usually between 4 and 10 years, with an incidence of approximately 3/1 million children and a double frequency in girls. Although of unknown etiology, jDM affects the skin (e.g., heliotrope eruptions, Gottron papules) and the proximal muscles progressively, often with systemic involvement (pulmonary, cardiac, gastrointestinal, endocrine). In addition, we cannot overlook the citation in the literature of SARS-CoV-2 as a trigger mechanism of jDM. Excluding the clinical-biological involvement specific to the pathology (also useful for confirming the diagnosis), Livermore P. et al. completes the symptomatic profile by highlighting the psychosocial manifestations identified among the patients. Their awareness is vital in the optimal, multifactorial approach to the consequences of the disease. It should be mentioned that the gold standard in diagnosis (muscle biopsy) is variably performed in children. The basic treatment used is an aggressive regimen of corticosteroid therapy, associated with immunosuppressants (Methotrexate, Ciclosporin, Cyclophosphamide, Azathioprine) or intravenous immunoglobulins. However, it is recommended to customize the therapy according to the patient's profile, following a "treat to target" approach. Biological agents are being studied (24–30).

### 2.2 Data on pulmonary involvement

Pulmonary function evaluation must be used both to refine the differential diagnosis and to dynamically monitor the severity of the disease. Early diagnosis allows targeted therapies to be instituted, thus maximizing results (31).

In addition to cardiac, renal or neurological damage, the lung damage identified in the case of jSLE is among the most diverse, bringing together both pleuropulmonary damage (pleurisy, pleurisy, acute lupus pneumonia, chronic interstitial lung disease, shrinking lung syndrome, pulmonary hemorrhages) and vascular (hypertension) pulmonary). Veiga CS. et col. note that approximately half of jSLE patients may present subclinical pulmonary abnormalities. The most frequent anomalies of the respiratory functional tests are the alteration of the carbon monoxide diffusion capacity and the total lung capacity. Its frequency is variable, being between 7% and 75%. On the other hand, the prevalence can register an upward curve, depending on the duration of the disease manifestation. However, Trapani S. et al. fail to identify a significant correlation between the changes in pulmonary function tests and the duration/activity of the disease or its immunological profile. Against them, recently, Dai G. et al. demonstrated the impact of both the duration of the disease and the immunological profile (positive anti-RNP and ANCA antibody) in dictating the risk of lung damage. After the research that involved 120 patients, Haupt HM. et al. attribute some of these changes to intercurrent infections, oxygen toxicity, or associated cardiac/renal damage (32–37). Consequently, pulmonary damage in jSLE is a vast field that lends itself to in-depth research due to its controversies.

Pulmonary involvement in jSSc is described by Murray KJ. et al. as being "a frequent, almost universal characteristic, if it is looked for carefully". Reports in the literature vary between 30% to 70%, noting an early involvement of the lungs in jSSc. Manifestations include interstitial lung disease, restrictive/obstructive pulmonary syndrome, pulmonary arterial hypertension (primary/secondary), and alteration of pulmonary function tests such as forced vital capacity or carbon monoxide diffusing capacity (17, 38, 39). Additionally, the medical world recognizes the association between SSc and malignant pathologies, including lung (e.g., adenocarcinoma), attributed in part to the genetic susceptibility of patients (40–42). In a practical way, since the specialized reports aimed at children are low in frequency, we emphasize the comparison obtained by Adrovic A. et al. between adult and juvenile disease phenotypes. Although the juvenile form seems to mainly affect the skin and joints, the authors objectify the presence of interstitial lung disease in a significant percentage of children (30%), compared to that of adults (50.9%). Thus, although rarely found in the clinical profile, possible lung damage in children with jSSc must be considered when diagnosing the pathology or in its evolution (21). To reinforce what was stated previously, the medical literature exposes cases of jSSc that are complicated by cardio-pulmonary damage (43, 44). Next, Adrovic A. et al. argue that it is the cardiovascular and pulmonary damage that largely dictates the prognosis of juvenile scleroderma (45).

Pulmonary involvement in jDM is a serious complication, but with a low prevalence. Manifestations include interstitial lung disease, aspiration pneumonia secondary to pharyngeal/esophageal dysmotility, and alveolar hypoventilation secondary to respiratory muscle weakness. The prevalence of DM symptoms depends on the spectrum of autoantibodies. Although lung damage is not correlated with the severity of the underlying condition, it negatively interferes with mortality. The importance of early diagnosis and treatment is therefore emphasized. Not to be neglected, despite its rarity, is the existence in the literature of cases with respiratory impairment due to CTD overlap syndrome. Popescu NA. et al. describes such a case of jDM-jSSc overlap. The importance of popularization lies in the need to add anti-PM/Scl antibodies in the diagnostic protocol (27, 46–49).

## 3 Pathophysiological mechanisms of lung damage

The heterogeneity of the pleuro-pulmonary damage in CTDs suggests divergent directions of the main pathophysiological mechanisms incriminated in the structural and functional decline of the respiratory system. Consequently, in what follows, we detail the general molecular lines of the degenerative process by correlation with the three entities that are the subject of the manuscript.

### 3.1 SLE

Although the etiology of jSLE is still open to research, it is certain that among the mechanisms involved in organic damage we find circulating immune complexes (CIC). The findings are supported by animal and human studies that reveal the involvement of autoantibodies in the pathogenesis of pulmonary complications. Quantitative research demonstrates an increased level of antibodies in lung tissue compared to circulating blood levels. Thus, CICs were isolated in alveolar septa, alveolar basement membrane, capillaries, large vessels, bronchi, and pleural effusion. Additional evidence is provided by the therapeutic efficacy of plasmapheresis (50–53). The latter represents an extracorporeal therapy that allows the removal of pathogens from the plasma. In the past, it was frequently used for complications such as renal or cerebral damage, thrombocytopenia, recurrent pulmonary hemorrhages, or antiphospholipid syndrome. Currently, use has been restricted to severe cerebral/pulmonary complications. Adverse effects were divided into mild in 2.4% of cases (related to the vascular approach in 54% and to the device in 7% of cases, in addition to hypotension and tingling), moderate in 3% (tingling, urticaria, hypotension and nausea) and severe in 0.4% of treatments (hypotension and syncope, urticaria, chills or fever, arrhythmia or asystole, nausea or vomiting). In addition to these, we note the risk of disturbing the coagulant and/or acid-base balance, infections, transfusion incompatibility, acute lung injuries related to transfusion and, respectively, the risk of diseases transmitted through the use of contaminated donor plasma. A report by Lu J. et al. which included 120 children with jSLE also noted a 2.5% incidence of toxic epidermal necrolysis after plasmapheresis. Consequently, although a well-tolerated procedure with excellent results in improving the general prognosis, among children prone to such complications, the risk-benefit ratio should be weighed judiciously. To increase the efficiency of the procedure, the clinician must consider the plasma volume (PV) that needs to be purified. It is calculated individually, according to the formula: PV = body weight (kg) × 0.065 × (1-hematocrit). It is estimated that to remove 75%-95% of a noxious substance, 1.4–3 PV must be changed during a procedure. To avoid the return of the pathogen in the blood circulation, the procedure must be repeated 4–5 times, at an interval of 24–48 hours (54–58).

At the molecular level, increased expression of genes regulated by type I interferon (IFN-1) is known. These are crucial in the process of promoting the synthesis of autoantibodies and deregulation of immune tolerance. Thus, pulmonary involvement may coexist with high levels of antibodies such as anti-double-stranded DNA (dsDNA), anti-La, anti-Scl-70, anti-SM, and anti-U1RNP, in contrast to decreased complement. Proinflammatory cytokines such as IFN-γ, tumor necrosis factor-α (TNF-α), interleukin-6 (IL-6), IL-8, IL-12, IL-17 also recorded high levels in patients with SLE that presented lung damage. At the opposite pole, IL-10 can be considered a protective factor for pulmonary manifestations in SLE. Other molecules involved in the pathogenesis of SLE are CXC chemokines. Among these, CXCL10 and CXCL11 are associated with the accumulation of neutrophils in the alveolar space, being therefore considered potentiating factors of interstitial fibrosis. Also, findings in cases with pulmonary fibrosis reveal overexpression of CX3C motif chemokine receptor 1 (CX3CR1) and CX3C chemokine ligand 1 (CX3CL1) (59–63). With regard to the pathogenesis of diffuse alveolar hemorrhages, during the research it was incriminated about its possible overlap with the COPA syndrome (mutations that damage the endoplasmic reticulum) (64). Despite the intense research in the field of molecular pathophysiology of jSLE, studies could not associate the changes of biomarkers with a pattern of clinical-imaging manifestations characteristic of lung damage from jSLE. Part of this limitation is attributed to the polymorphic manifestations. They could not allow the formation of sufficient study groups to be able to issue findings. Next, Ding H. et al. note the potential of using biomarkers in the development of precision medicine in SLE (65, 66). We encourage the concentration of future efforts towards the deepening of research in the field. We strongly believe that they could bring a new perspective in the anticipation of the evolutionary pattern and the personalized management of children with jSLE.

### 3.2 SSc

In SSc, lung damage mainly follows two directions similar to the basic pathology, represented by fibrotic and immuno-inflammatory pathways. In addition, we consider the endothelial dysfunction that is considered as a bridge between the immune and fibrotic damage. This is triggered by the autoimmune attack doubled by the influence of genetic and environmental factors. Although apparently common with those of idiopathic interstitial lung disease, the pathogenic pathways followed in autoimmune diseases are delimited by the dynamics of their involvement (e.g., divergent IFN signaling patterns). Summarizing, alveolar, and microcirculatory injury can be considered the trigger factors both in the activation and proliferation of lung-resident immune cells, as well as in the recruitment of inflammatory cells (e.g., monocytes, neutrophils, mast cells and natural killer cells). To these is added as an aggravating factor the dysregulation of microvessels and the regeneration capacity of lung progenitor cells (type 2 alveolar cells). Subsequently, pro-fibrotic mediators modulate the activation of the Toll Like 4 receptor (TLR4), promoting the uncontrolled, excessive deposition of extracellular matrix and potentiating transforming growth factor-β (TGF-β) signaling. The finality of the process is illustrated by epithelial and endotheliosis mesenchymal transition, promoting the transition of fibroblasts to activated myofibroblasts and extensive lung remodeling, culminating in the irreversible disorganization of the lung structure, increased stiffness and functional impairment. Consequently, the beneficial effects of modulating the TLR4 pathway in the management of pulmonary fibrosis are suggested. Autoreactive B lymphocytes also participate in this cascade, by favoring the secretion of pro-fibrotic mediators and autoantibodies. The pro-inflammatory balance is particularly supported by neutrophil-derived alpha-defensins (human neutrophil peptides), cytokines and chemokines (IL-8, IL-1α, IL-10, CCL2, CCL7, CCL18, macrophage inflammatory protein-1α and MCP- 1). An inverse correlation was also observed between lung function and myeloid-derived suppressor cell (MDSC) levels, thus attesting to their potential involvement in the pathogenesis of pulmonary fibrosis (67–74). Other pathways and molecules recently described as being involved in the balance of pulmonary fibrosis associated with SSc are: Wnt/β-catenin signaling, Yes-associated protein (YAP) and transcription coactivator with PDZ-binding motif (TAZ), nuclear receptor subfamily 4A (NR4A), CXCL4, Sirtuin1, matrix metalloproteinases, cathepsins or endostatins (73, 75–77).

### 3.3 DM

The causative factors of jDM appear to be an entanglement of genetic susceptibility (e.g., HLA-DQA1 * 0501, HLA-DQA * 0301, HLA-DRB1 * 0301) with disturbing environmental factors. The major pathogenic event is complement-mediated damage to vessels, triggering additional cytokine release via the membrane attack complex. From this point, an inflammatory cascade centered on IFN-1 leads to major histocompatibility complex class I (MHC I) overexpression and dendritic cell (DC) maturation. Downstream appears the regulation of adhesion molecules, the modulation of lymphocyte migration and the inflammatory infiltration of the muscles. Further, the extensive studies on IFN-1 suggest the involvement of the cytokine in the relation of anti-melanoma differentiation associated gene 5 (MDA5) autoantibodies - interstitial lung disease (ILD) within the JDM subtype characteristic of them. The importance of knowing and recognizing the amyopathic form of DM to which we refer (DM5) resides in its high prevalence in pediatrics (10–40% of jDM cases) and the rapidly progressive evolution, with high early mortality, of ILD. In this case, the indications for therapy with aggressive corticosteroids are controversial, the only one noted being the decrease in the possibility of progression to classic jDM. After associative therapy, anti-MDA5 antibodies may become negative. The genetic predisposition is equally felt. Thus, the genotype-phenotype correlation indicates HLA-DRB1*03 as a predictive factor of the risk of ILD, independent of the presence of autoantibodies. To all this is added the possible trigger effect of RNA virus infections (e.g., coxsackie, parvovirus B19, SARS-CoV-2) (27, 78–81).

The pathological mechanism of pulmonary murmur in dermatomyositis is complex and still open to research. Regarding ILD, CD8 T lymphocytes are thought to exert a diffuse cytotoxic effect on muscle cells, while B lymphocytes and CD4 T lymphocytes (predominant in perivascular areas) may be responsible for muscle vasculitis. B cells also seem to be correlated with disease activity, being able to be used both as a biomarker and as a therapeutic target. This theory was supported by the lung biopsy results that revealed numerous CD8 lymphocytes both in the affected tissue and in the partially preserved tissue. Also, their percentage in the bronchoalveolar lavage fluid could be correlated with the response to corticosteroid treatment. Pulmonary arterial hypertension (PAH) occurs rather as a consequence of ILD, being partly precipitated by vasoconstriction secondary to chronic hypoxia and vascular remodeling (78, 82).

**Figure 1:**
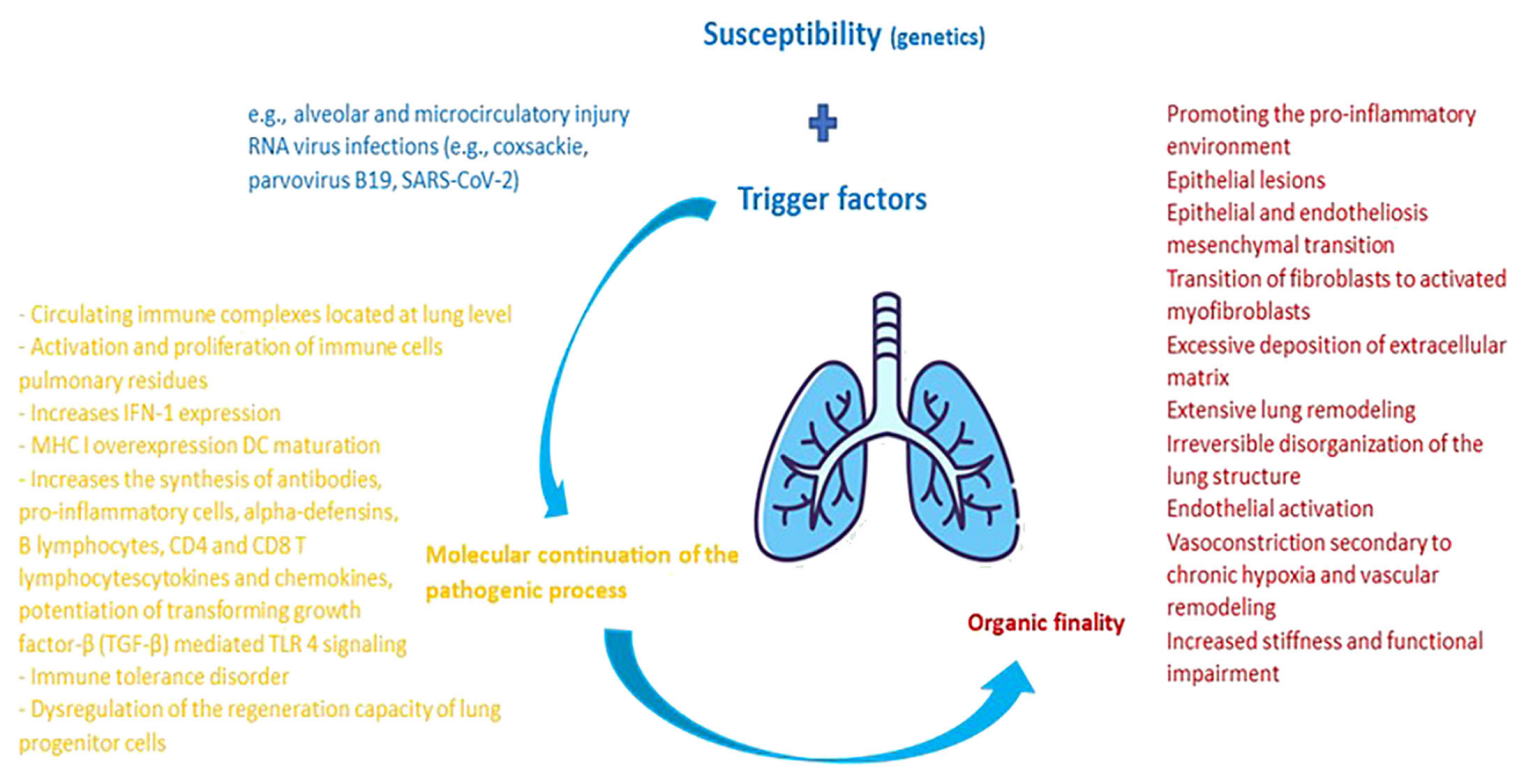
Pathophysiological cascade characteristic of pulmonary damage from collagenoses. The main factor of pulmonary damage from collagenoses is presented by genetic susceptibility. External triggers are additionally added to it. Upon contact of an organism genetically predisposed to the development of a collagenoses with external factors that potentiate the pulmonary injury, an activation of the host's immune system takes place. The molecular balance at this level maintains the pathological process, having the practical purpose of inflammation and damage to the adjacent pulmonary and vascular parenchyma. In dynamics, the lesions heal through remodeling and fibrosis, thus imprinting pulmonary elasticity and organic functionality.

Summarizing the previously developed directions, **Figure 1** shows the main pathological mechanisms underlying the pulmonary damage in collagenoses, respectively jSLE, jSSc and jDM.

## 4 Types of pulmonary damage

Pulmonary damage in CTDs is diverse, gathering under its umbrella pathologies such as pleurisy, pleural effusion, acute pneumonia, emaciated lung syndrome, interstitial lung disease, diffuse alveolar hemorrhage, pulmonary arterial hypertension, and pulmonary embolism. Although the clinical profile and the diagnostic method are broadly similar, the division of the diseases in terms of frequency is dependent on the collagenoses pattern.

### 4.1 Pleurisy

Pleural involvement in lupus is one of the most frequent consequences of the disease (approximately 80% of jSLE cases), being included in the classification criteria. Further, pleurisy notes a frequency of 12.5–32%, being able to be complicated by the appearance of pleural effusion. The localization can be unilateral or bilateral, of small to moderate amount and with the possibility of recurrence. We can rarely find the existence of the "dry" form of pleurisy. Clinically, it can evolve from an asymptomatic phase to one manifested by chest pain accentuated by inspiration, dry cough, fever and dyspnea. The diagnosis must be confirmed by chest X-ray or high-resolution computed tomography (HRCT). The balance is later completed by measuring the C-reactive protein (CRP), the erythrocyte sedimentation rate (ESR), lactate dehydrogenase, thrombomodulin and the pleural puncture which reveals a sterile, yellow serous liquid, with a predominance of polymorphonuclear neutrophils or lymphocytes. Immunologically, the high level of antinuclear antibodies (>1/160) is a potent indicator of the involvement of SLE in the pleural etiology. In the absence of SLE as etiology, the high level of antibodies frequently indicates paraneoplastic causes. Finally, susceptibility to pleuropulmonary lesions is suggested by leukopenia, low complement C3 fraction or dsDNA antibodies (+) (32, 50, 83–85). Pleural effusion in scleroderma is rarely reported in the literature, both in the pediatric and adult populations. However, its occurrence has recently been correlated with PAH within CTDs or, more rarely, with the coexistence of Meigs syndrome (recurrent pleural effusion, ascites and often benign ovarian tumor formation) in adults. This aspect can partly explain the interest in using elevated serum CA125 values as a marker of serositis in CTDs (86–89). In jDM, pleural damage is rarely found, the exudative effusion can appear isolated or in association with the pericardial effusion (78).

### 4.2 Acute lupus pneumonia

It is relatively rare in the evolution of jSLE, and may also be the initial manifestation of the disease, as presented by Şişmanlar Eyüboğlu T. et al. The frequency noted in its case is approximately 11% of patients. Histologically, diffuse alveolar lesions, interstitial edema, formation of hyaline membranes and even hemorrhagic areas can be observed. Clinically, it is defined by non-specific signs such as fever, cough, dyspnea and cyanosis due to hypoxia. Consequently, the factors that induce a correct diagnosis are the onset of ALP in correlation with the exacerbation of the underlying disease involving other organs, doubled by the positivity of anti-SSA antibodies in most cases. In dynamics, it may appear necessary for fan support. Imaging we observe pulmonary infiltrates in the presence/absence of pleural effusion. Important in the management of ALP are the diagnosis and countermeasures of associated infections and pulmonary hemorrhage, entities that can mimic the clinical profile. Because infections are the leading cause of death in patients with SLE, the diagnostic workup must include appropriate cultures, doubled as needed by fiberoptic bronchoscopy, transbronchial biopsy, and open lung biopsy. Therapeutically, the administration of immunoglobulins must be carried out with caution due to the risk of precipitation of renal lesions (32, 50, 90–92).

### 4.3 ILD

ILD within CTDs can currently be divided according to the histological characteristics into usual interstitial pneumonia, non-specific interstitial pneumonia, desquamative interstitial pneumonia and/or respiratory bronchiolitis-associated interstitial lung disease, organizing pneumonia, lymphocytic interstitial pneumonia, pleuroparenchymal fibroelastosis and alveolar lesions loudspeaker (93). Studies on ILD in jSLE are few. However, studies that included adults demonstrated a weak ILD-SLE association in the absence of overlap of other autoimmunity (94). The characteristic damage of jSSc begins with the accumulation of a mixed cellular infiltrate (bronchoalveolar lavage positive for neutrophils and eosinophils) in the pulmonary interstitium that will overflow into the alveolar spaces, constituting the first, inflammatory phase. The image of ground-glass opacities on the HRCT of the lungs is suggestive at this stage. Progressively, the inflammation decreases, being replaced by a transition towards thickening of the alveolar walls, fibrosis and pulmonary remodeling. Moderate restrictive lung disease/severe decrease in lung volumes develops consecutively. Clinically, it is mainly objectified slowly progressive dyspnea on exertion and productive cough. Other suggestive aspects in evolution on HRCT are reticular linear opacities, honeycombing, nodules, cylindrical bronchiectasis, and parenchymal bands. Associations between esophageal damage and respiratory dysfunction in children with jSSc have also been described. An accompanying complication can be aspiration pneumonia following esophageal dysmotility associated with jSSc (17, 32, 95–97). It should be mentioned that chest x-rays do not show pathological aspects except in the late stages of ILD, thus being included in the category of insensitive diagnostic methods. Likewise, repeating the bronchoalveolar lavage is not justified, since no post-therapeutic changes were objective (95). In conclusion, we emphasize the strong correlation between ILD, and mortality encountered in SSc. Castelino FV. et al. expose, with the help of a cohort of adults, the precocity of the development of restrictive lung disease independent of individual characteristics (age, gender, duration of the disease) and the importance of screening in the population at risk (98). In agreement, Hoffmann-Vold AM. et al. underlines the need to create some classifications of ILD from the perspective of severity and the risk of progression, but also the importance of early screening. The desired purpose is the optimization and individualization of therapeutic schemes to increase the quality of life (99).

ILD in jDM has been reported to affect approximately 8% of patients. The most common forms described are nonspecific interstitial pneumonia and cryptogenic organizing pneumonia. Among the early markers of interstitial disease in CTDs, we note Krebs von den Lungen-6 (KL6), expressed by damaged type II alveolar cells. IL-19 and ferritin (>1000 ng/ml) are added to this. The correlation between KL-6 and IL-18 suggests the possible association of alveolar macrophages in the pathogenesis of ILD. At the same time, anti-aminoacyl transfer ribonucleic acid synthetase (ARS) antibodies, rare in JDM patients, are associated in a proportion of 63% with ILD in juvenile idiopathic inflammatory myopathies. Clinically, the symptomatology is non-specific, dry cough and respiratory difficulties being described. Spontaneous pneumothorax and pneumomediastinum are not frequently encountered, probably due to vasculopathy. However, respiratory function tests, including carbon monoxide diffusing capacity, should be evaluated at diagnosis and in dynamics (79, 84, 100–103). ILD has also been reported in anti-MDA5 jDM. The described pattern includes skin, mucous and joint damage, in addition to the pulmonary component. Contrary to adults, the rapidly progressive evolution was variable, also describing a favorable therapeutic response compared to the anti-MD5 negative form. However, there are also citations in the literature of fatal cases of IDL with negative anti-MDA5. Compared to the general form, the clinical profile of jDM5 can be complicated by pneumomediastinum in 15% of cases, a condition clinically characterized by increased dyspnea, cervical or facial swelling, subcutaneous emphysema, cervical pain, and cough. It has a negative impact on mortality, especially in cases with non-invasive ventilation in the therapeutic scheme. Early recognition, the establishment of optimal treatment, as well as screening in risk populations are measures that lead to improving the prognosis dictated by lung function (78, 79, 104–106). For this purpose, Hu M. et al. defined, with the help of a cohort of 93 children, a nomogram based on variables such as ESR, IL-10 and MDA-5 antibodies. This has proven effective in the clinical evaluation and prediction of long-term prognosis of ILD associated with jDM (107).

### 4.4 Chronic interstitial lung disease

CILD in patients with SLE is characterized by inflammatory cell infiltrate in various degrees, peribronchial lymphoid hyperplasia, homogeneous interstitial fibrosis and pneumocyte hyperplasia. In particular, the medical literature describes the existence of lymphoid interstitial pneumonia, characterized by infiltration of the interstitium with polyclonal lymphocytes and pneumocyte hyperplasia, along with moderate macrophage and lymphocytic alveolitis. Clinico-biologically, pulmonary function is affected similarly to diffuse infiltrative pneumonia. Radiologically, fibrosis is rare (50). Furthermore, it is estimated to affect approximately 14% of children with jSLE. The clinical-imaging presentation is marked by signs and symptoms such as chronic cough, dyspnea, exercise intolerance, fatigue, or pleural effusion. The diagnostic gold standard is HRCT (ground glass appearance or evidence of fibrosis) due to the reduced sensitivity of chest X-ray. Given the chronic and minimally symptomatic nature of the condition, the negative imprint of pulmonary function is less often objective and more difficult to interpret (32).

### 4.5 Shrinking lung syndrome

It is a rare manifestation but cited in the literature of jSLE. From an etiological point of view, the means of its occurrence is unclear, being dysfunctions or hypo functions of the diaphragm (unilateral or bilateral) due to muscle or nerve damage (phrenic nerve). The clinical pattern of SLS includes progressive, episodic dyspnea with orthopnea, hypoxia, pleural pain aggravated by inspiration, pleurisy, a restrictive pattern of lung volumes, with/without parenchymal imaging abnormalities (frequently, however, atelectasis may be present). Percussion reveals dullness in the lower lung lobes, characteristic of reduced expansion. SLS usually appears months to years after the diagnosis of SLE. Chest X-ray shows small lungs, without pleuropulmonary damage (except for atelectasis). Additionally, for diagnostic purposes, the use of diaphragmatic ultrasound in M mode or fluoroscopy is recommended. The response to early optimal therapy can be favorable. Consequently, we emphasize the importance of including SLS in the category of differential diagnoses of dyspnea in children with SLE (32, 50, 108–111).

### 4.6 PAH

PAH is defined by the progressive increase in vascular resistance and obstructive vascular remodeling, which over time leads to increased mortality. PAH is currently considered a pulmonary vascular disease with systemic noise. Recently, the possibility of distinguishing between the idiopathic form of PAH and that associated with scleroderma has been postulated based on the changes evident in the level of circulating bioactive metabolites (e.g., acid metabolites, eicosanoids/oxylipins, sex hormone metabolites). The pathogenesis is multifactorial bringing together, among others, inadequate angiogenesis, inflammation/vascular obstruction, metabolic/immune disorders, DNA damage, genetic mutations, and impairment of vasoreactivity. Initial evaluation can be performed with the help of echocardiogram. The condition often appears 1.5 years after the initial manifestations of CTDs. The gold standard in diagnosis is represented by right heart catheterization. The technique can measure elevated mean pulmonary arterial pressure (>25 mmHg at rest) by reference to normal pulmonary capillary pressure (<15 mmHg). The prevalence in the pediatric population with SSc is lower compared to adults (prevalence estimates <10%). Accordingly, children with PAH showed a lower risk of heart failure compared to adults, although the risk of syncope is higher. Current medical literature ranks SLE as the second cause of PAH precipitated by CTDs, after SSc. The risk factors for PAH in jSLE are Raynaud's phenomenon and the presence of antiphospholipid antibodies, although Anuardo P. et al. did not objectify a PAH pathogenic correlation in jSLE and the antiphospholipid antibody syndrome. At the opposite pole, the prognostic factor considered important is PaO2. Optimal and early management has improved life expectancy. In pediatrics, it mainly addresses congenital heart defects, as there are no specific recommendations specifically addressed to cases associated with scleroderma. Therapeutic combinations (e.g., Bosentan and Iloprost/Sildenafil) require further studies. A viable alternative seems to be lung transplantation (17, 32, 50, 84, 95, 112–115). PAH in DM is rare in the absence of ILD (78).

### 4.7 Alveolar hemorrhage

AH occurs most frequently as a consequence of pulmonary microcirculation damage, being categorized as one of the emergencies in jSLE. It occurs most frequently in girls, in cases with previously diagnosed SLE, being often associated with lupus nephritis. In this context, the suspicion arises that corticosteroid therapy for kidney damage is among the precipitating factors of AH. Of all the pulmonary manifestations, AH is marked by an acute onset with hemoptysis, anemia, and diffuse pulmonary infiltrates on chest X-ray. To these can be added cough, fever, dyspnea, hypoxia, asthenia, tachycardia, increased inflammatory markers, positive direct Coombs test, thrombocytopenia, and consumption of complement factors. Radiologically, it is presented by diffuse infiltrates. The pulmonary diffusing capacity of carbon monoxide is increased, the bronchoalveolar lavage (the key investigation in the examination) demonstrating the presence of blood or hemosiderin-laden macrophages. The biological consequence is a significant decrease in hemoglobin. Simultaneously, immunology described the presence of immune complexes and IgG and C3 deposits in alveolar, capillary, and interstitial cells. The incidence and mortality are high in the pediatric disease, being clearly higher than that reported in adults. Management requires an aggressive approach, centered on mechanical ventilation, therapy with corticosteroids, immunosuppressants, intravenous immunoglobulin or plasmapheresis depending on the needs (32, 50, 84, 116–119). Recently, such episodes have been correlated in the medical literature with acute respiratory infection caused by the SARS-Cov-2 virus (120).

### 4.8 Pulmonary thromboembolism

It can be found especially among adolescents with jSLE, being precipitated by smoking, estrogen-based contraceptives and proteinuria resulting from nephrotic disease. There are described in the literature cases of pulmonary thromboembolism as the first symptom in jSLE, from the investigation of which the diagnosis of the underlying disease was started. It is mainly a complication of the coexistence of antiphospholipid antibodies, especially the lupus anticoagulant. Pulmonary thromboembolism can be found especially among adolescents with jSLE, being precipitated by smoking, estrogen-based contraceptives and proteinuria resulting from nephrotic disease. There are described in the literature cases of pulmonary thromboembolism as the first symptom in jSLE, from the investigation of which the diagnosis of the underlying disease was started. It is mainly a complication of the coexistence of antiphospholipid antibodies, especially the lupus anticoagulant (50, 84, 121–126).

## 5 Management methods

In closing the manuscript, we would like to emphasize the importance of multidisciplinary monitoring in the dynamics of patients with CTDs. This must be carried out optimally, including clinical examination and paraclinical investigations (biological/imaging), mainly due to the risk of overlap in the evolution of pathologies. Argumentatively, we bring to attention the report submitted by Lin HK. et al. They describe the case of a 15-year-old child (known to have SSc) in whom, following the investigations initiated by the systemic decline of the underlying condition, the coexistence of the diagnostic criteria for SLE and pulmonary involvement (serositis, fibrosis and PAH) is objectified (127). The literature in this regard is vast, also recognizing cases of triple association of pathologies in dynamics (SLE, DM and later SSc) (128). In such situations, we should not lose sight of the possible involvement of microbiome disturbances in the imprinting of the risk of development, association or aggravation of pathologies, as described in the literature and in case of other diseases (e.g., atopies, autoimmunities, gastrointestinal or renal pathologies) (129–136).

Also, taking JSLE as an exemplary model, patient monitoring must be done gradually, starting with primary care doctors who must recognize the diagnostic/alarm signs of the pathology and continuing with rheumatologists, experts in the field of pathogenesis, pediatricians, cardiologists, orthopedists, and other medical categories that can be skilled in the management of complications. In the work team, an essential role is occupied both by clinical pharmacologists, competent to anticipate and prevent possible drug interactions, and psychotherapists. Good staffing leads to optimized therapeutics by early recognition of disease complications, minimizing drug toxicity, educating families about prevention, promoting school performance, addressing reproductive health concerns, and easing the transition to health care of the young adult (137). Chang JC. et al. notes that children who benefited from a multidisciplinary approach recorded better levels of the pediatric index of care, compared to those who benefited from monodisciplinary follow-up (138). Being a chronic pathology, current studies have also focused their attention on the barriers regarding good therapeutic practices. Among these, we mainly note the patient's lack of compliance due to psychological, systemic degradation or the deficit in primary education, the brutal transition between the team of pediatric specialists and that of adults, non-involvement/non-information of the patients regarding the natural course of the pathology and management methods, material shortages, biases of the medical system regarding financing, communication and the collaboration of specialists and social stigmatization. Looking at the pediatric-adult transition, a recent study showed that this period is characterized by moderate disease activity, partially precipitated by the emotional attachment to the previous medical team, doubled by a prolonged time until being noticed by the new medical team (139, 140).

Summarizing the previously presented data and supplementing them with other references from the literature, **Table 1** shows the main lines in the recognition and management of pulmonary disorders in CTDs in children. We thus outline a clinical guide that summarizes the correlation between the type of collagenoses and the frequency of pulmonary injury, the paraclinical investigations (biological and imaging) that must be undertaken to certify a correct diagnosis and effective management means, currently certified. Additionally, we review the particularities of the conditions and the pharmacological substances used, where appropriate, but also the biases and future directions in the individualized therapy of the affected child.

**Table 1:** Practical clinical guideline in the recognition and management of pulmonary lesions in CTD in children - current and future trends (adapted from Ramphul M. et al., García-Peña P. et al., Lammi MR. et al., Landini N. et al., Buda N. et al., Sperandeo M. et al., DeCoste C. et al., Shimizu M. et al., Eyraud A. et al. and Shin JI. et al.) (84, 141–149).

Affection	Specificity of CTDs	Paraclinical investigations	Imaging features	Management directions	Remarks
Pleurisy	jSLE +++	leukocyte CRP, ESR, LDH, thrombomodulin thoracentesis + biochemical analysis ANA CA125	CXR/HRCT: unilateral/bilateral pleural effusion, in small to moderate amounts, with opacification of the costdiaphragmatic recess	Analgesics and anti-inflammatories Oxygen therapy Steroid therapy (Methylprednisolone) Tetracycline pleurodesis	
	jSSc +				
	jDM +				
ALP	jSLE ++	Positive anti-SSA antibodies Histology: diffuse alveolar lesions, interstitial edema, formation of hyaline membranes, hemorrhagic areas spirometry	CXR/HRCT: pulmonary infiltrates in the presence/absence of pleural effusion	Steroids Immunosuppressive Immunoglobulins ^3^ Plasmapheresis Antibiotic therapy	Bacterial cultures, fiberoptic bronchoscopy, transbronchial biopsy, and open lung biopsy are recommended to rule out associated lung infections
	jSSc -				
	jDM -				
ILD	jSLE +	ESR BAL ^4^: positive for neutrophils and eosinophils KL6, IL-10, IL-19, IL18, ferritin ARS MDA5 spirometry, DLCO Gastrointestinal evaluation	CXR: changes only in late phases HRCT: ground-glass opacities, reticular linear opacities, honeycombing, nodules, cylindrical bronchiectasis, parenchymal bands, esophageal dilatation Ultrashort echo-time MRI TUS	Steroids, Immunosuppressants (Hydroxychloroquine, Azathioprine, Methotrexate, Cyclophosphamide, Mycophenolate mofetil, Infliximab, Rituximab) Oxygen therapy Lung transplant/autologous hematopoietic stem cells Prokinetic agents	Immunosuppressive agents require in-depth studies regarding the effectiveness in children In refractory cases, Adalimumab or combined therapy with high doses of methotrexate, cyclosporin A and intravenous immunoglobulins can be used Nintedanib is currently showing promising results in adults
	jSSc +++				
	jDM ++				
CILD	jSLE ++	Histological: inflammatory cell infiltrate in various degrees, peribronchial lymphoid hyperplasia, homogeneous interstitial fibrosis, pneumocyte hyperplasia, macrophage/lymphocytic alveolitis spirometry, DLCO	HRCT: pleural effusion, ground glass or fibrosis appearance TUS		TUS can provide information about pulmonary damage in the initial stages, being non-invasive and useful in assessing the need for HRCT
	jSSc no				
	jDM no				
SLS	JSLE +	Blood gasometry Spirometry	CXR: small lungs, atelectasis, high diaphragm HRCT: atelectasis diaphragmatic ultrasound mode M: alteration of the dynamics of the diaphragm fluoroscopy: alteration of the dynamics of the diaphragm	Corticosteroids Theophylline and beta-agonists may be useful in diaphragmatic weakness	The syndrome is rare, the therapeutic schemes being incompletely developed. Rituximab demonstrated its effectiveness in the study on isolated cases
	jSSc no				
	jDM no				
PAH	jSLE ++	Echocardiography: right ventricular enlargement Right heart catheterization Antiphospholipid antibodies Blood gasometry	CXR, HRCT, MRI: the increase in size of the pulmonary artery, explores the existence of congenital defects	Steroids Immunosuppressants (Cyclophosphamide) Vasodilator (Bosentan, Epoprostenol, Iloprost/Sildenafil) Lung transplant	Bosentan can induce hepatic dysfunction as an adverse effect pentraxin-3 (PTX-3) is a biomarker under investigation regarding the effectiveness of estimating the risk of SSC-PAH
	jSSc +++				
	jDM + ^1^				
AH	jSLE +++	Hemoglobin, Hematocrit, Platelets Coagulogram, Coombs test Serum complement Inflammatory markers: ESR, CRP Blood gasometry, Spirometry, DLCO Echocardiogram: tachycardia BAL: blood/macrophages loaded with hemosiderin Immunohistological: immune complexes and deposits of IgG and C3 in alveolar, capillary and interstitial cells	CXR: diffuse pulmonary infiltrates MRI Bronchoscopy	Mechanical ventilation Corticosteroid therapy Immunosuppressants (Cyclophosphamide, Rituximab) Intravenous immunoglobulin Plasmapheresis	
	jSSc no				
	jDM no				
PT	jSLE +++^2^	Complete blood count Coagulogram Antiphospholipid antibodies	CTPA: may show pulmonary embolus Doppler: may show venous embolus	anticoagulants Thrombolysis	
	jSSc no				
	jDM no				

* +++, frequent; ++, relatively rare; +, rare; -, absent; no, no data; jSLE, juvenile systemic lupus erythematosus; jSSc, juvenile systemic scleroderma; jDM, juvenile dermatomyositis; ALP, acute lupus pneumonia; ILD, interstitial lung disease; CILD, chronic interstitial lung disease; SLS, shrinking lung syndrome; PAH, pulmonary arterial hypertension; AH, alveolar hemorrhage; PT, pulmonary thromboembolism; CRP, C-reactive protein; ESR, erythrocyte sedimentation rate; LDH, lactate dehydrogenase; ANA, antinuclear antibodies; BAL, bronchoalveolar lavage; KL-6, Krebs von den Lungen-6; IL-, interleukin; Ig, immunoglobulin; C3, C3 fraction of the complement; DLCO, diffusing capacity of the lungs for carbon monoxide; ARS, anti-aminoacyl transfer ribonucleic acid synthetase antibodies; MDA5, anti-melanoma differentiation associated gene 5 autoantibodies; CXR, chest x-ray; HRCT, high-resolution computed tomography; MRI, magnetic resonance imaging; TUS, thoracic ultrasound; CTPA, computed tomography pulmonary angiogram;

^1^in the absence of ILD;

^2^in association with antiphospholipid antibody syndrome, smoking, estrogen-based contraceptives and proteinuria;

^3^caution due to the risk of precipitating kidney damage;

^4^dynamic repetition is not recommended.

## 6 Conclusions

In conclusion, CTDs represent a topic of interest in pediatrics, the approach of which requires the formation of multidisciplinary teams composed, among others, of rheumatologists, pulmonologists, cardiologists, pediatricians, physio/kinetotherapists and psychologists. Although they differ in some respects from the clinical pattern found in adults, they are not without damage at a systemic level. Treatment can broadly include immunosuppressive drugs, corticosteroids, and other therapies designed to control inflammation and relieve symptoms. Given the burden on the quality of life, psychotherapy sessions can be necessary and useful. Focusing on the pulmonary damage, we reiterate that the damage to the respiratory system can be multiple, both at the pleural, interstitial, and vascular levels. At the same time, the functional impairment can also be found in children without suggestive pulmonary imaging changes. Therefore, we consider that the current work has achieved its defined objective by promoting the increase of information, diagnosis, and optimal management among children with collagenoses. The value of the work is given by the mirror approach to respiratory damage in the three frequent pathological entities (SLE, Sc and DM), both from a physiopathological and a management point of view. Also, through the final subchapter we bring to light both fundamental knowledge, already postulated in the literature and current research directions in the field. In the end, we reiterate the importance of screening in at-risk populations to reduce the burden of subsequent comorbidities and increase the patient's quality of life and his capacity for social integration.

## Author contributions

AL: Conceptualization, Investigation, Writing – original draft. MS: Project administration, Validation, Writing – review & editing. EJ: Investigation, Methodology, Writing – original draft. AA: Investigation, Software, Writing – original draft. MA: Investigation, Software, Writing – original draft. IS: Validation, Visualization, Writing – review & editing. AM: Investigation, Software, Writing – original draft. AN: Validation, Visualization, Writing – review & editing. AK: Software, Validation, Writing – review & editing. DS: Software, Validation, Writing – review & editing. SB: Validation, Writing – review & editing. VL: Methodology, Supervision, Writing – review & editing. II: Investigation, Methodology, Writing – original draft.

## Glossary

**Table:**

AH	alveolar hemorrhage
ALP	acute lupus pneumonia
ANA	antinuclear antibodies
ANCA	antineutrophil cytoplasmic antibodies
anti-RNP	anti ribonucleoprotein needle
anti-SM	anti-Smith
ARS	anti-aminoacyl transfer ribonucleic acid synthetase
BAL	bronchoalveolar lavage
C3	C3 fraction of the complement
CIC	circulating immune complexes
CILD	chronic interstitial lung disease
CL	chemokine ligand
CR	chemokine receptor
CRP	C-reactive protein
CTDs	connective tissue diseases
CTPA	computed tomography pulmonary angiogram
CXR	chest x-ray
DC	dendritic cell
DLCO	diffusing capacity of the lungs for carbon monoxide
DM	dermatomyositis
DNA	deoxyribonucleic acid
dsDNA	anti-double-stranded DNA
ESR	erythrocyte sedimentation rate
HRCT	high-resolution computed tomography
IFN	interferon
Ig	immunoglobulin
IL	interleukin
ILD	interstitial lung disease
jDM	juvenile dermatomyositis
jLSc	localized juvenile scleroderma
jSc	juvenile scleroderma
jSLE	juvenile lupus erythematosus
jSSC	systemic juvenile scleroderma
KL6	Krebs von den Lungen-6
LDH	lactate dehydrogenase
MCP	monocyte chemoattractant protein
MDA5	anti-melanoma differentiation associated gene 5
MDSC	myeloid-derived suppressor cell
MHC	histocompatibility complex
MRI	magnetic resonance imaging
NR4A	nuclear receptor subfamily 4A
PAH	pulmonary arterial hypertension
PaO2	partial pressure of oxygen in the arterial blood
PT	pulmonary thromboembolism
Sc	scleroderma
SLE	lupus erythematosus
SLS	shrinking lung syndrome
TAZ	transcription coactivator with PDZ-binding motif
TGF	transforming growth factor
TLR	toll like receptor TNF- α, tumor necrosis factor-α
TUS	thoracic ultrasound
YAP	Yes-associated protein
